# "The Hospital" Nursing Mirror

**Published:** 1898-02-26

**Authors:** 


### "The Hospital" Nursing Mirror.

Being the Nursing Section of "The Hospital."

[Contributions for this Section of "The Hospital" should be addressed to the Editor, The Hospital, 28 & 29, Southampton Street, Strand, London, W.C., and should have the word "Nursing" plainly written in left-hand top corner of the envelope.]

### News from the Nursing World.

### JUBILEE MEMORIAL AT NICE.

The Queen will probably enjoy the Jubilee celebration at Nice, which is timed to take place during her visit. A new ward, named the "Victoria," which has been built, furnished, and, to the extent of one bed, endowed, will then be opened in her honour, possibly by some member of the Royal Family.

### THE CZARINA.

The Czarina is suffering from a slight attack of measles, nothing serious however. She is not sufficiently indisposed to render it necessary to issue bulletins.

### DEATH OF ANOTHER NURSE OF THE PLAGUE.

We are sorry to record the death of another nurse from plague. Upon hearing that two lady nurses at Bombay were suffering from the plague we made inquiries at the India Office as to how they were progressing, and have received in reply a copy of a telegram from the Governor of Bombay, dated the 23rd inst., running as follows: "We regret to say that lady nurse Harriet McDougall died of plague at Bombay yesterday evening."

### NURSES IN THE FRONT.

Lady nurses attached to the British General Hospital in the Punjab will remain on duty there until active operations cease and the hospital is closed.

### THE QUEEN OF PORTUGAL AND MEDICINE.

A delightfully interesting paragraph to the effect that the Queen of Portugal was studying medicine with a view to treating her husband has unfortunately been shown to be untrue. M. Cabenès, a journalist, culled the tale and wrote to Her Majesty, asking for further particulars! He received a reply that not only had the Queen never studied medicine, but that she was not doing so now and merely took a humanitar ian interest in the subject!

### THE KEOGH PRIZE.

A considerable sum raised amongst the medical staff and students of Guy's Hospital in memory of Nurse Keogh has been expended, partly in placing a tablet to her memory in the chapel of the hospital, and partly in founding a "Keogh Prize," to be awarded yearly to the nurse probationer obtaining the highest marks in the surgical examination. The prize will take the form of a surgical dressing-case.

### OPENING OF NEW BUILDINGS AT PORTSEA.

In consequence of the death of the Duchess of Teck, the Duke and Duchess of York were unable to open the new hospital, infirmary, and nurses' home in connection with the Portsea Island Workhouse. The ceremony was therefore performed by the Mayoress of Portsmouth on the 22nd inst. The total cost of the new buildings is £24,000.

### MISS FRANCES WILLARD.

Miss Frances Willard, whose death we are sorry to see announced on the 17th inst., was a woman of world-wide renown. Celebrated as the instigator and prime organiser of the Woman's Temperance Union, she had gifts that would have raised her to eminence in letters had she devoted herself to their cultivation. She was born of parents of high character, and nurtured in rude simplicity in the backwoods of America. Beginning life as a teacher, she lived and died a teacher, but of very different scholars to her first girlish pupils. She was a delightful woman, sympathetic, amusing, eloquent, and strenuously in earnest. Her logical mind speedily grasped the uselessness of legislation not upheld by moral suasion; and her tongue, her pen, her time were alike devoted to the crusade against strong drink. Miss Willard, who has died at the comparatively early age of 59, was beautiful, being possessed of the domed head and chiselled features that are usually associated with the ideal of the Madonna.

### THE PUNJAB UP-COUNTRY NURSES.

Owing to a slight misapprehension on the part of our correspondent with regard to the Punjab branch of the Up-country Nursing Association we were led to state that candidates should be Protestant ladies. This. however, was an error. We learn that there is no religious test, and ladies of high character and good nursing qualifications are eligible, irrespective of creed.

### NOT CLEVER ENOUGH.

The circumstances mentioned in the paragraph entitled "Too Clever by Half," which appeared in last week's Hospital, supply an amusing instance of the danger to a woman editor of allowing prejudice to make her jump to conclusions. In her endeavour to be sharp, the editress has not even been careful. She has caught at the shadow and overlooked the substance. As a matter of fact it was unnecessary to drag in a deceased member of the staff, for Mr. G. B. Hudson, M.P., is himself actively connected with the Middlesex Hospital, being an energetic member of its committee of management. We do not see what the Middlesex Hospital has to do with the letter Mr. Hudson wrote to the *Lancet*, but we have no doubt the *Nursing Record* will now start a series of articles showing the enormity of this connection, its dangers to the whole nursing profession and to women throughout the world, and generally call upon Parliament and Providence to protect both from the ruin which awaits them if this nefarious conspiracy is not nipped in the bud.

### DISTRICT NURSING IN LINCOLNSHIRE.

Amidst the controversial clashing of opinions as regards the best way of nursing the poor comes the excellent record of the Lincolnshire Nursing Association, of which Lady Winchilsea is the president. Two distinct grades of nurses are employed, the fully trained nurse and the rural maternity nurse who is thoroughly trained in midwifery. The cost of the former is from £60 to £80, and of the latter from £35 to £45, and both are under the inspection of Queen Victoria's Jubilee Nurses' Institute, with which the association is affiliated, and Miss Peter's report of their work is very good. The second part of the association's work, namely, the training of nurses, is carried on carefully and successfully, and is aided by the Nursing Scholarships granted by the County Council. There is also an annual meeting for the nurses (delayed we regret to see on account of the illness of both Lord and Lady Winchilsea), and a nurses' library. The association now covers 20 districts employing 27 nurses; an increase of seven nurses and three districts during the year. Districts are always encouraged to employ the more fully trained nurses as being both more satisfactory and economical, whilst those who cannot afford it, have at any rate the second best, namely, a woman trained in midwifery and under constant supervision.

### NURSING IN VANCOUVER.

St. Luke's Home, in Vancouver, shows a very good record for the last twelve months. The six nurses on the staff have had plenty of hard work, but they have also had the satisfaction of seeing their work growing and appreciated. Sixty-four cases altogether have been nursed, of which number 16 were nursed gratuitously, 25 paid full fees, and the rest what they could afford. Interesting patients crop up, too, such as a gentleman on the road to Klondyke, and a lady doctor on her way to the Corean Court; so that, however far away Vancouver may be, it is certainly on one of the world's highways betwixt East and West. The new Victoria Ward attached to the home for Englishmen—the token of rejoicing at the Queen's Diamond Jubilee—is finished, but not completely furnished. When this is full of paying patients, and the home is more widely known, a season of prosperity will have set in which is now only foreshadowed. Strangely enough, the residents of Vancouver have been the most laggardly contributors, a state of affairs that is, we are glad to note, not to be allowed to continue. Free from debt, new developments safely launched, recognised by the Queen, St. Luke's Home may be said to have done very well indeed during the auspicious year of 1897.

### A MORIBUND CHARITY.

It is the oddest thing to observe how people forget even a good thing if it is not kept constantly before them. Here is a case in point. At Woodley, near Reading, consequently within easy reach of town, there is a small convalescent home, which has accommodation for nine or ten men. In spite of the house being there, ready furnished in a healthy and convenient spot, the local committee have recently closed it chiefly because there is a falling off of patients. Mr. F. Wilder, of Purley Hall, near Reading, will gladly receive communications from anyone willing to undertake to reopen and manage the home. Considerable local subscriptions may be relied on, and certainly the ostensible reason for closing it, namely, that there was a falling off of patients, is one that would speedily cease to exist if the home were properly made known.

### A WOMAN'S HOSPITAL ON MEDICAL WOMEN.

Several medical women were candidates for appointment as resident medical officers for 1898 in the Sydney Hospital. After some discussion and complimentary remarks about the work done in Melbourne by medical women the appointments were given to medical men.

### THE CARE OF THE SICK IN THE RIVIERA.

It is so common now for delicate folk to rush southwards as soon as winter's chills make themselves felt that the British Hospital at Sunny Bank, Cannes, fills a want that is frequently keenly realised. Sir Sydney Waterlow has shown energy and discretion in adapting and organising the hospital for the reception of patients. He has had the buildings overhauled and added to, the drainage brought up to the standard of modern sanitation, facilities arranged for the isolation of infectious cases, and resident medical and nursing staffs appointed. In providing this fully-equipped hospital the resources of the organisers have been exhausted, but it is hoped that residents and visitors of this famous health resort will subscribe liberally to its support. Those who use it will know how to appreciate the advantages of having every help in illness at hand, and those who do not require it ought surely to remember it in their charities as a suitable object to which to devote their thankofferings.

### SHORT ITEMS.

Mrs. Walsh, wife of the Bishop of Mauritius, intends, if possible, to build a hospital for the native women. In the event of this charitable project being carried out, the hospital will be placed under the charge of a woman doctor, as the patients have the same objection to medical men as the Indian women.—The Frome Home for Trained Nurses Committee, after 14 years' work, is seriously meditating a removal into a cheaper house. The extra receipts for the Jubilee have enabled the committee to straighten their accounts and begin the new year with a balance of £6; but the contemplated removal would ensure a saving of £25 a year. Unless something turns up, either in the way of handsome subscriptions or an equally substantial reduction in the rent and taxes of the premises at present occupied, the proposed change of residence will take place in due time.—The report of the Southampton Nurses' Institute shows it to be active and prosperous, in spite of a deficiency of £43 in the current accounts. There are 14 nurses of the staff, and the difficulty of procuring good nurses has confirmed the committee in the practice of training their own.—The annual report of the Southport and Birkdale District Nursing Society is a favourable one. The accounts are strictly kept, the administration expenses are light, and it has been so well supported that £50 has been transferred to the suspense fund. The invalid kitchen worked in connection with the society is not self-supporting, owing to the fact that the dinners sent out to the patients are paid for by the society. A sixth nurse has been added to the staff during the year.—The villages of Bourn, Caxton, Papworth, Everard, Eltisley, Croxton, Waresley, and the two Gransdens, on the borders of Cambridgeshire and Huntingdonshire, have formed a branch of Cottage Nursing Association in connection with the Holt Ockley system.—Barnstaple is to have a parish nurse. She will be engaged for one year, and the time will be prolonged if subscriptions come in to warrant the expenditure.—Another district nurse is to be added to the Bedfordshire Trained Nurses' Institute. —Two English nurses of the Bombay General Hospital have been attacked by the plague.—Nurse Pickersgill, parish nurse at York Road, was presented, for the most part by her poor patients, with a carriage clock, barometer, thermometer, and compass, all combined, in a leather case. She has been nurse at York Road for three years, and is leaving to take up work at Helston, in Cornwall.

### Lectures to Surgical Nurses.

By H. A. LATIMER, M.D. (Dunelm), M.R.C.S. of Eng., L.S.A. of London, Consulting Surgeon, Swansea Hospital; President of the Swansea Medical Society; Lecturer and Examiner of the St. John Ambulance Association, &c.

### XXV.—DESCRIPTION OF BED SORES—METHODS FOR THEIR PREVENTION AND CURE.

Now let me describe a bed sore from its earliest appearance to its full development. Starting as a dusky red discoloration of skin, unless this state is quickly remedied by appropriate treatment, this damaged skin breaks, and, peeling off the under lying tissues, exposes a raw spot. The little wound rapidly enlarges, and what was at first a dark red sore of the size of a quarter of an inch, quickly becomes three or four times as big; it also excavates the muscles, so that it spreads downwards as well as outwards. The ulcer now formed is a foul dirty one, with ragged edges and a surface of sloughing tissues, and, if unchecked in its progress, may extend right down to the bones. I have seen, but fortunately very rarely, huge bed sores of this kind in which the devitalised area attacked by sloughing denuded a greater part of the lower great bone of the back. Such a condition of gangrene as this is an absolute proof of the want of knowledge, and of care, to which the poor sufferer has fallen a victim; for, even when the process has started, it is possible by the application of well-known principles of treatment to arrest its progress, and to bring about a healthier condition of affairs. Let me sketch for you the measures which are to be adopted for prevention and cure of these bed sores, laying great stress upon the former part of my subject. First, as to the bed on which the patient lies. This should consist of a mattress covered by a blanket and a sheet; on no account should it consist of a yielding material into which the patient can sink into inconvenient pits, such as is the case if a feather bed is used. I extract a paragraph from one of Clark Russell's novels—"The Lady Maud"—a paragraph which exactly describes the best kind of bedding for everyone, if it can only be obtained: "The science of upholstery hit upon the most perfect bed for comfort, rest, and refreshment when it designed the spring mattress, and the hair mattress on top of it." Great care should be taken to smooth out the blanket and sheet and to tuck them in at the mattress so that they may not become "rucked up" in places, and so act irritatingly to the patient. But even these great precautions are oftentimes insufficient in prostrate conditions, and then a water bed must be secured on which the patient may lie, this buoyant and yielding resting-place allowing of an equalisation of pressure on the body, and so preventing any one part of it being especially selected for damage. A word or two of caution is necessary about this appliance. Be careful to see that it is filled with warm water at about the temperature of the body, and that it is tested before use to make sure of its not leaking. The men who bring it to the house will do this for you. I remember one case where the water bed was filled with cold water, with unhappy results. Sometimes air and water pillows of circular or horseshoe shapes are used, but although they are of great service in taking off pressure from pressed-upon places, they cannot compare in comfort to the water-bed.

You notice that the essential principle of prevention and of cure is that of removing pressure on the skin at parts liable to be damaged. Another way of applying this principle, besides the one I have spoken of, is by not allowing the invalid to lie in one position for a long time. In low and prostrate conditions the circulation of the blood is carried on feebly by a weakened heart, and there is a great tendency for it to stagnate at the most dependent points, setting up the morbid condition known as "hypostatic congestion." This hypostatic congestion quickly passes into inflammation, with all the untoward consequences of that malady, and as "prevention is better than cure," a valuable part of a nurse's duties consists in making the patient shift his position from time to time so that the blood-vessels may be relieved of stagnating blood, as well as the burden being taken off pressed-upon parts of the body. If the invalid is not strong enough of himself to move about in bed you will move him, when lying helpless, more comfortably and easily with the draw-sheet than you will with your hands. This draw-sheet is placed on the top of the under-sheet and consists of a double-fold of cotton sheeting extending from the patient's shoulders to below the hips. It serves a double purpose—guarding the under-sheet from being soiled by discharges, and enabling the attendants to roll and turn the patient lying upon it without straining or discomforting anyone. If the nurse wants to turn her patient, she grasps this sheet with each hand at its corners, having previously turned aside the upper bed-clothes, and lifts it upwards. The patient's body is now raised lengthways, and all she now has to do is to see the body is kept on the side on which it now lies by pillows if it is to remain in this position, or by someone's hands if the change of position has been made only for some temporary purpose, such as attending to the back. We will for the moment assume that this position has been gained for this last purpose. The nurse now looks carefully to see whether there are any evidences present of pressure anywhere—should there be, they will betray themselves by the dusky red or bluish-red skin which I have spoken of. If she hastens to take all pressure off the threatened area of skin by air-cushions or a water-bed, and rubs this skin twice a day with a spirit lotion composed of equal parts of eau de Cologne and water, or brandy, or whiskey and water, she will restore it to a healthy condition, and will prevent the bed sore forming; but if she lets this precious opportunity pass by, in a very short time the skin will break and a sore will form. The rubbing with the spirit lotion does two things; the spirit hardens the skin and renders it resistant to injurious influences, and the rubbing diffuses the stagnant blood and promotes its free circulation through the blood vessels.

By constant vigilance on your part you should be able to prevent the formation of bed sores in all conditions of prostration other than in paralytic states, where the tissues slough as the direct result of withdrawal of nerve influence. Here, sometimes, they will appear in spite of every care. Your greatest trouble will be to ward them off from invalids who are suffering from leakage of the bladder or of the bowel. There are lots of indiarubber and china appliances for using in such conditions of incontinence, but in spite of all your care you will find them to be ineffectual in preventing contamination of the bed sheets. In such cases you must constantly examine the patient and his bedding, and remove soiled draw-sheets directly they are to be discovered to be in that condition, wash the back, dry carefully, rub with spirit lotion, and dust freely with some antiseptic powder such as powdered boracic acid or powered oxide of zinc, and then, after carefully smoothing out the new draw-sheet and underlying sheets, replace the patient in a comfortable position. If the skin has broken, and this is the whole extent of the mischief, you should apply zinc ointment containing camphor. A good formula for this ointment is 30 grains of camphor to one ounce of zinc ointment, which has been prepared with vaseline instead of lard.

### Nursing in Paris Hospitals.

### A.—LAY NURSES.

III.—Engagements, Dismissals, and Transfers.

Until a few months ago the lay nurse has been engaged on application at the hospitals, there being no seeking of nurses by the Assistance Publique. Hitherto the municipal officials have taken such material as was offered to them, and the choice has not been of a very superior sort.

The administration of the Assistance Publique has realised the fact that the indiscriminate recruitment at the door of the hospital needed a thorough amendment, and on July 1st, 1897, when an entirely new scale of wages came into force, a new method of engagement was ordered. These subjects were regulated by two successive circulars issued by M. Peyron, the chief of the Assistance Publique of Paris, on June 25th, 1897. The first circular gave details (which I will treat of presently) of the new wage scale, conformatory to the decision of the Municipal Council in December, 1896. The new wages were ordered to date back to January 1st, 1897. The second circular, regulating the new method of engagement of nurses, was substantially as fellows: (1) The grade of assistant nurses, housemaids, and porters will henceforth include two categories—1st, the probationary male and female nurses, housemaids, and porters; 2nd, the first and second class assistant nurses, housemaids, and porters. (2) The probationers will be recruited by the directors of the establishments. They will be submitted within eight days of their admission to a medical examination to see if they are fit and are exempt from contagious infections. If the result is favourable they will be admitted for a probation of not less than six months, at the expiration of which they may become assistant nurses, on the express condition that during that period they have not only given complete satisfaction to their chief, but that the probation has been passed in the same establishment and without interruption. Those found unsatisfactory must be sent away within eight days. (3) The probationers, in addition to all allowances, will receive wages at the rate of 350 francs per annum, payable monthly. (4) The grade of assistant employé will be conferred by, and can only be withdrawn by, the Director of the Assistance Publique. (5) All assistant employés who leave the establishment where they are engaged without the permission of the Director of the Assistance Publique will lose the benefit of their grade. They cannot be readmitted into any establishment under the Assistance Publique except as probationers, and under the same conditions as new recruits.

The circular concluded with an announcement that the employés then in the hospitals who had benefited by the augmentation of wages detailed in the other decree would have a right to be classed amongst the assistant nurses, and that they would be hereafter subject to the same regulations as those employés admitted and promoted after the new rules have been put in force. The genesis of the new regulations is part of a new development in the ranks of the nurses themselves, and which I will describe later on. Though there was doubtless a desire in many quarters to improve the quality of the nursing staff, a good deal of the inspiration of the new circular came from a growing determination among the employés to make some effort towards rendering their situations less precarious; and, of course, it is always a great point with all wage servants to diminish as much as possible the supply of substitutes for themselves from the great unemployed out of doors, thus making themselves more masters of the situation. The more difficulties put in the way of recruiting new nurses, and the more hospital chiefs are hampered by regulations both in this respect and in the power of discharging the present employés, the more the majority of the employés are likely to be pleased.

Of course, it is essential that to a great extent the director shall be master in his own house, but it will be seen that under the new rules the director can neither absolutely engage nor absolutely discharge a duly commissioned nurse. The director's power over engaging or discharging probationers remains absolute, but as to commissioned nurses he can only recommend either an engagement or a dismissal. In the case of engagements the director's recommendation is an essential factor, and in the case of dismissal, of course, his recommendation is generally acted upon, if due reason is given.

There is another and more surprising form of recruitment of nurses for the Paris hospitals which I will more fully touch upon in discussing the character of nurses. This is the engagement of patients in the hospitals on the cure of their maladies, and even before the maladies have been entirely effaced. This practice is so surprising, so essentially objectionable, and so much to the front just now in Paris as a subject for a radical reform, that I will throw in my next article all the light I can upon the question.

The reasons for dismissals and resignations are also largely bound up with the question of character, but I will give the following figures of the departure of nurses from the service of the great Lariboisière Hospital from July 1st, 1895, to July 1st, 1896: At their own request, 117; to other hospitals, 11; on promotion, 3; resigned, 1; military service, 5; refused by central administration, 11; for drunkenness, 2; for negligence, neglect of duty, 31; for insubordination, 45; death, 3; total 229. The figures of La Pitié during the same period, giving an interesting separation of the sexes, are as follows: On their own demand, men 28, women 19; illness, men 9, women 8; transfers, men 13, women 22; refused by administration, men 5, women 4; dismissed for idleness, negligence, men 9, women 8; death, men 1, women 1. The Hôtel Dieu, which is still under the Sisters, shows generally a much better record, there being only 61 in 1894-5, 66 in 1895-6, and but 47 in 1896-7. It must be noted that the Hôtel Dieu is larger than La Pitié, and nearly as large in accommodation as Lariboisière. The respective stability of the nursing staff I will, however, touch upon in discussing the question of comparative merit of the religious and lay administration.

The hospital chiefs have hitherto been so much bothered with the frequent changes in their lower grade nurses that they hail the new rules as a protection against the tendency of new nurses to resent anything like discipline, and to go off in a huff to some other hospital when taking any offence. As every wine shop near a hospital is an employment information agency, a new job has been quickly obtained by those who knew how to go about it. Now the new rule about continual service in one hospital for probationers will check this. On the other hand, the upper nurses have for years been demanding a regular scheme of frequent transfers of nurses for education and experience, and also that preference should be given in re-engagements of nurses to those who have had to leave for "serious reasons, such as sickness or marriage."

EDMUND R. SPEARMAN.

### A Private Nursing Staff on New Lines.

MRS. STRONG, Matron of the Glasgow Royal Infirmary, who was the originator of the system now embodied by the London Hospital in its Tredegar House scheme, has inaugurated another new departure in nursing. In the middle of January, being desirous to do the best she possibly could for the nurses trained at the Royal Infirmary, Glasgow, Mrs. Strong found another alternative outlet for them to that of staff or charge nurse. Nurses who have been trained at the Royal Infirmary are placed upon a list for private work, and it is notified that their services can be obtained on application to the Matron. The terms are 8s. per day, £2 2s. per week; the charge for attendance and operations is one guinea, and all the fees are paid to the nurse without deduction. It has been found to work well, and there are already eight nurses attached to this staff; and it is proposed to increase the numbers as the demand arises. So far, no advertisements have been issued, the nurses being principally used by the physicians and surgeons attached to the infirmary. The only payment made by the nurse is 1s. per case, which covers the cost of telegrams and postage. There are no expenses, and all the office work is done without charge by Mrs. Strong. When disengaged the nurses live in their own homes or in lodgings. They take what holidays they please. They report themselves to the matron when they wish to take a case, and there are no printed regulations of any kind. As more nurses are required the matron puts up a notice; and she holds herself responsible for the behaviour and conduct of all nurses supplied for private work. Each nurse is at liberty to take cases on her own account, providing she intimates to Mrs. Strong when she is so engaged. So far the scheme has been successful. We shall watch the working of this new departure with great interest, and we are sure that the thanks of every one are due to Mrs. Strong for having initiated it. No doubt should the number of nurses be greatly increased steps will be taken to relieve Mrs. Strong of much of the incidental labour, not only for her own sake, but in order to ensure the permanence and smooth working of the new system.

### Wants and Workers.

[THE attention of correspondents is directed to the fact that "Helps in Sickness and to Health" (Scientific Press, 28 & 29, Southampton Street, Strand, London, W.C.) will enable them promptly to find the most suitable accommodation for difficult or special cases.]

MISS E. A. HANCOCK, 156, Anerley Road, would be glad to have a second-hand copy of Burdett's "Hospitals and Charities" for 1897.

MISS SCHNEIDER, Freie Strasse, 34, Zurich, will be glad to receive back copies of the "Nursing Mirror," and will pay the carriage if any nurse having them at her disposal will write to her.

THE matron of a small convalescent hospital would be so glad of an old piano for the use of the patients, and for concerts, &c. She would pay carriage, or a small sum for it if necessary.

### The Art of Fault-Finding.

LOOK upon fault-finding as an art, not a science. Make it a system, and the result is grey, dull, and monotonous; to be really effective it requires a light touch here, a dark smirch there, by a master hand who knows the precise value of each wordy stroke and can appreciate every shade of intonation.

Doubtless it would be a pleasanter world if there were no occasion for finding fault, but as the world is at present the opportunities are but too frequent. It may seem a strange idea, but really the study of this art is an essential part of our training—we learn it subjectively that we may apply it objectively. While we were probationers we became practically aware that no day passes without illustrations; the right way seemed as narrow as the needle's eye. We had then ample opportunity for observing the different methods of fault-finding, and discovered them to be as various as the weather. In one ward the systematic grumbling has been so constant we almost ceased to notice it; here was no ray of encouragement, and we dwelt in an atmosphere of chilly dampness and hopelessness. Then again, we have experienced wards where the air was charged with electricity, where we were shouted at and storms burst with little or no warning. This made us nervous, and we broke more thermometers and treasures of the "doctor's table" than usual. Or, perchance, we may have found ourselves enveloped in fog, a kind of groping in the mist, feeling we were in disgrace, but unable to discern the reason. Other wards were characterised by breeziness and sudden squalls, and we sailed in anxious uncertainty whether the shifting wind would blow with or against us. Possibly some of us may have encountered the pitiless rain of relentless severity, stinging words of rebuke that made us bow our heads and clench our teeth. In short, most of the fault-finding has been depressing, and made us feel our life "a weary, weary river" flowing to "the bitter, barren sea."

Now, why cannot this unfortunately necessary adjunct of training be made bracing and invigorating by means of a spontaneous expressiveness that, while it makes the culprit tingle and smart, at the same time exhilarates her and gives her an appetite for her work? It is so dull to be constantly snubbed and growled at, while to be stormed at and blown up, or treated with icy disdain, is to be disheartened without being improved. But take an erring probationer and treat her as if she were a human being like unto yourself, tell her her fault incisively, in vivid words and picturesque language, covering the point of your remarks with a foil of delicate satire or touch of humour, and ten to one she goes away and wonders over what you have said, and the moral you have meant to enforce gradually dawns upon her and so imprints itself on her memory. In this way it may be even possible to make an impression upon the feather-bed-style of person whose stolid demeanour refuses to yield to coarser forms of treatment. The method may not always be crowned with success; some people may only think you "very sarcastic," but on the other hand, like bread cast upon the waters, you may find your words returned after many days, proving that at least they have been considered and remembered.

### Where to Go.

THE secretary of the Royal British Nurses' Association requests us to give notice that the sessional lecture "The Nursing of Europeans on the West Coast of Africa," by Miss Mary Kingsley, announced for Friday, February 25th, has been unavoidably postponed till Tuesday, March 8th, owing to the illness of Miss Kingsley.

### London Temperance Hospital.

OPENING OF THE NEW NURSING HOME.

For a long time the nurses at the London Temperance Hospital have cast longing eyes at one of the houses facing the back of the hospital, which appeared so desirable as a nurses' home. Through the generosity of Mr. and Mrs. George Cadbury, the premises are now theirs, and on Wednesday the donors held an At Home to open the new home. The house selected has been completely renovated. A handsome dining-room has been built, and a connecting passage between the home and the hospital. The building is of two stories, and contains six charming bedrooms, a drawing-room, a nurses' kitchen, and a bath-room. The bedsteads are of black and brass, and combination dressing chests and wardrobes of light polished wood, long mirrors, easy chairs, and occasional chairs are found in each bedroom. The dining and drawing room are for the use of all the nursing staff, numbering 30. The drawing-room is provided with comfortable chairs and couches, writing tables, and a piano. The dining-room, which is a handsome apartment, is comfortably and suitably arranged, and has a pleasant outlook upon a public garden. Above the dining-room is a flat roof, which has been converted into a winter garden, and here the nurses hope to take tea in the summer. The nurses of the Temperance Hospital are to be congratulated on this charming home, given to them by Mr. and Mrs. Cadbury in memory of their mother, one of the earliest friends of the hospital. Later a tablet to her memory will be placed in the entrance of the home. The building has been furnished entirely by Messrs. Maple, who have carried out the wishes of the donors in the most satisfactory manner.

### Presentations.

At Craig House, the private patient department of the Royal Edinburgh Asylum, on February 16th, Nurse Watson was presented with a clock and two bronze ornaments by the staff on her leaving after nearly seven years' service. The presentation took place in the newly-furnished nurses' recreation room. A short time ago another popular member of the staff, Miss Elkins, was given a gold bracelet by the staff on her leaving for hospital training.

Miss Agnes E. Bourne, on leaving Chalmer's Hospital, Edinburgh, was presented with two handsome gifts. From the directors and secretary she received a silver hot water jug of old-fashioned pattern, and from her nurses a prayer-book. She also received a letter regretting her departure and saying how much her work as matron for the last four years had been appreciated.

Miss Clara H. Bourne, who has been matron of the Royal Chest Hospital, City Road, for the last five and a half years, was presented, on leaving, with a silver salver of handsome design by ten ward sisters and nurses. The gift was accompanied by a letter expressing great regret at her resignation.

Southwold Diamond Jubilee Cottage Hospital.—Miss Eliza Payn has been appointed Matron of the above hospital. She received her training at St. Mary's Hospital, Paddington, and has since had much experience in surgical home and private work, both medical and surgical.

### Minor Appointments.

Essex and Colchester Hospital.—Miss Jarman has been appointed Sister at this hospital. She was trained at St. Mary Abbotts Infirmary, Kensington, and has held the post for one year as charge nurse at the South-Western Hospital under the Metropolitan Asylums Board.

The Leeds House of Recovery and Convalescent Home, Gildersome.—Nurse E Mellor has been appointed Sister to the above home. She has just finished training at Bagthorpe Fever Hospital, Nottingham.

### The Book World for Women and Nurses.

[We invite Correspondence, Criticism, Enquiries, and Notes on Books likely to interest Women and Nurses. Address, Editor, The Hospital (Nurses' Book World), 28 & 29, Southampton Street, Strand, London, W.C.]

A Manual of Nursing: Medical and Surgical. By Laurence Humphry, M.A., M.D., Assistant Physician and formerly Lecturer to Probationers at Addenbrooke's Hospital, Cambridge. With numerous illustrations. Pp. 244. Sixteenth edition. Price 3s. 6d. (Charles Griffin and Company, Limited, Exeter Street, Strand. 1897.)

The fact that this useful little work has reached its sixteenth edition is proof of the popularity it still maintains with the public. For long it was the only text-book of any considerable merit adapted to the needs of nurses. The classification of various diseases and the management of each in regard to nursing are excellent, and will always be found of the greatest value in deciding doubtful points where the nurse is left to her own judgment. Chapter I. treats of the general management of the sick-room. The importance of its aspect, temperature, ventilation, and furniture are all dealt with in turn, with suggestions as to what is best in each case. We could have wished, however, for a little more detail on one or two points which at present are somewhat inclined to be vague. For instance, in the matter of water-beds, it would have been as well to mention the test by which a nurse judges when it is sufficiently filled with water, and we could have wished that among the preventives of bed sores, the simplest but most efficacious of all, viz., well washing the back with a soapy lather night and morning, had found a place. The directions for changing sheets and the use of sick-room appliances are both clear and practical; but we must demur unhesitatingly to resorting under any circumstances to the insanitary and objectionable habit of rolling up the soiled part of a draw-sheet and tucking it under the mattress. Chapter II. gives an admirable outline of anatomy, and affords a basis for the subsequent chapters. Chapter III. describes the nervous system and its principal diseases. Among the most useful chapters in the book are those devoted to diseases of the respiratory and digestive systems, and the hints given in respect of their nursing leave little to be desired. The management of infectious fevers will be found of great value to those engaged in attendance on such cases. The directions as to isolation and disinfection are explicit and in accordance with the best hygienic principles. The average quantity "of half a pound of sulphur or more according to the size of the room" is perhaps rather small for disinfecting purposes, few rooms being of less capacity than 1,000 cubic feet. This, however, is a minor detail, and does not in any way detract from the usefulness and value of the book which Dr. Humphry presents us with in a new and enlarged form.

### Appointments.

MATRONS.

Barrow-in-Furness Workhouse Infirmary.—Miss Maud M. Wray was elected on February 16th Superintendent Nurse of this infirmary. She was trained at the Liverpool Training School and the Royal Infirmary and Home for Nurses. She has since been charge nurse for Fir Vale Infirmary, Sheffield, and superintendent nurse of the City of London Infirmary.

The Gordon Road Workhouse, Peckham.—Miss Annie Beverley, who was trained and afterwards staff nurse of the Middlesex Hospital, was appointed Superintendent Nurse of this workhouse on February 16th. After obtaining her certificate Miss Beverley was engaged for a year in private nursing.

Chesterfield Workhouse Hospital.—Miss Sarah J. Macbeth, who was trained at Chorlton Union Infirmary, was appointed Superintendent Nurse of this infirmary on February 19th, 1898.

### Everybody's Opinion.

[Correspondence on all subjects is invited, but we cannot in any way be responsible for the opinions expressed by our correspondents. No communication can be entertained if the name and address of the correspondent is not given, as a guarantee of good faith but not necessarily for publication, or unless one side of the paper only is written on.]

### NURSE ATTENDANTS.

We regret that the want of space will not permit the insertion of another letter upon this subject. Doubtless if "An Invalid" communicated her wishes to a superintendent of a reliable nurses' home she would endeavour to meet them.

### THE WARWICK LADIES' CHARITY.

The Countess of Warwick writes: I notice a letter signed "Justice" in The Hospital for February 12th, and, as President of the Warwick "Ladies' Charity," would like to point out to you the rule to which "Justice" refers: "Every woman receiving a ticket will be required at the end of the month, if sufficiently recovered, or as soon after as possible, to go to the parish church, or any other place of worship, and return public thanks to Almighty God for her recovery; to have her child baptised at the same time, or sooner, if necessary." You will thereby see that the charity is undenominational, the woman being required to attend her own "place of worship."

### TRIALS OF A MONTHLY NURSE.

"A Perplexed Monthly Nurse and L.O.S., Rotunda," writes: The L.O.S. unfortunately does not insist on a good lying-in hospital certificate showing the practical fitness of the midwife for her work. The great part of a midwife's training should be learning to know not only her own work in normal labour, but when something abnormal is likely to occur, and when to send for the doctor in time to prevent rather than help her out of a difficulty. Surely it is only in the wards and labour wards of large hospitals where one can see and watch the many cases, before and after the birth of the infant, that this can be learnt. It is so easy to know what to do or not to do in theory, so utterly different when it comes to practice. I advise the perplexed L.O.S. holder to go to Queen Charlotte's Hospital or the Rotunda, or any large lying-in hospital, and she will understand the great difference between the L.O.S. and a hospital certificate.

### PUERPERAL FEVER IN ACHILL ISLAND.

Nurse Blackmore, a Queen's Nurse of Irish Branch, writes: I can confirm the information contained in your columns respecting the prevalence of puerperal fever in Achill Island. So bad was it a few months back that no less than five young mothers one after another succumbed to this fell disease. The congested Districts Board took the matter up, and they are paying for a Queen's Nurse—a trained midwife—for the island. At first she did not get on, but we are able to say now that she has just nursed a puerperal fever—her first—case back to convalescence. Her fame has now gone abroad, and the people say she can cure every disease. The day of sending in her monthly report a woman had travelled across the island, walking several miles with a huge child, who was sick, in her arms to consult nurse, because she had heard "the nurse could cure everybody." Nurse battles with the "wise woman," and the traditional remedies, *e.g.*, a man's waistcoat has to be worn, be it never so dirty, by the lying-in woman, and a man must be in the room during the accouchement, and many other marvellous things has the nurse had to overcome, and with half the cabin given up to the lord and ladies of the land—cows, bulls, and ponies—not forgetting the gentleman what pays the "rint." It is, indeed, in and out of season that the nurse has not only to preach but put in practice what she teaches. At the nurse's first case—a confinement—there were four cows and a donkey and the doctor's horse, four men and three women—a cabin is not even a good sized room—and there was the doctor and the nurse; but these are only some of the small matters to overcome. When nurse has to travel over six or eight miles of bog and mountain in a hurricane off the Atlantic, you can imagine she does not quite enjoy the fœtid atmosphere of the cabin. Yes, even in the wilds of Achill is one of our sisters waging war with disease, dirt, and ignorance, which we hope she will abolish.

### MISS FREEMAN'S RETIREMENT.

Miss A. M. Jennings writes: Would you allow me through the medium of The Hospital to ask any old pupils of the British Lying-in Hospital, Endell Street, who would like to express their friendship and respect for Miss Freeman on her retirement, to send their names and a small subscription to Miss Jennings, care of Secretary, Midwives' Institution, 12, Buckingham Street, W.C. It has been found impossible to communicate with all privately, but we know there are many who would be sorry not to have the opportunity of joining us.

### HOW TO DIAGNOSE CAUSE OF DEATH.

"Nurses C. and Mc." write: We the undersigned charge nurses of the above infirmary, have a friendly difference of opinion, and which we both most respectfully ask you to decide for us, as to the similarity or otherwise at the autopsy of death by hanging, strangulation, drowning, choking, inhaling poisonous gases, viz., miners' choke damp, charcoal fumes, coal gas, or any such things and substances as fatally interfere with healthy breathing. We both agree that outward appearances on such bodies as to where and how found would help the pathologist to diagnose cause of death at the autopsy, all of which signs we wish you to cloak or disregard and merely give an internal diagnosis: 1. Totally ignoring these ocular outward symptoms for the sake of our argument, which we, of course, agree are inseparable, would the post-mortem reveal the mode of death? 2. Would the blood and internal organs be alike in each case in any or either at death? 3. Would the cause of death in each and every case be suffocation? 4. Would the post-mortem reveal the mode, form, or kind of suffocation? We presume all these persons to be similar in health previous to such a sad fate.

*** We hardly think that it is fair to make use of our columns to decide what looks very like a sort of wager! Nevertheless, as the questions put seem to show an interest in their work, which we are glad to encourage among all nurses, we will offer a few remarks on the matters involved. For full particulars a book on medical jurisprudence should be consulted, but it will be evident that the conditions found in the heart and lungs after death in cases where the breathing has been obstructed will be very different from those discovered where death has taken place suddenly from syncope, that is, from primary cessation of the heart. Now in strangulation, choking, many cases of drowning, and in suffocation by carbonic acid gas, the obstruction is in the lung, and the condition of heart and lungs is much the same in all. But there is this difference, that in drowning there will usually be water in the bronchial tubes, and often, indeed, mud and particles of water weeds and other substances in the upper air passages, pointing without doubt to the cause of death. In choking, again, the bit of food that has caused the mischief will often be found in the larynx or trachea, while in strangulation there will be signs of bruising in the neck. Drowning, however, does not always cause death by suffocation. Sometimes a deadly syncope takes place at once, and death takes place by the heart rather than by the lungs. Again, charcoal fumes are not always of carbonic acid; often they consist of carbonic oxide which does not kill by suffocation, but by poisoning of a very peculiar sort, which leaves definite traces in the condition of the blood after death. The same applies to miners' choke damp. Sometimes this consists principally of carbonic acid, but often, and especially where the explosion has resulted from coal-dust rather than from coal-gas, the final product is carbonic oxide, which produces its own peculiar symptoms. Hanging also does not always kill in the same way. Sometimes—generally in cases of death by the hangsman—the neck is broken and death is instantaneous; but in suicides and clumsy hangings the death is caused by suffocation and has its signs. Thus it is generally possible by post-mortem examination—even taking the internal signs alone—to discover the cause of death. But this is attained not by any rough rule, but by a consideration of all the appearances met with.

### For Reading to the Sick.

"WHEN THOU PASSEST THROUGH THE WATERS I WILL BE WITH THEE."

**Verses.**

MEN as men
Can reach no higher than the Son of God,
The Perfect Head and pattern of mankind.
The time is short, and this sufficeth us
To live and die by; and in Him again
We see the same first starry attribute,
"Perfect through suffering," our salvation's seal,
Set in the front of His Humanity. . . .
While we suffer, let us set our sons
To suffer perfectly; since this alone—
The suffering—which is this world's special grace,
May be perfected and left behind.
—*H. Hamilton King.*

The path of sorrow, and that path alone,
Leads to the land where sorrow is unknown.
—*Cowper.*

We did amiss when we did wish it gone
And over; sorrows humanise our race;
Tears are the showers that fertilise this world;
And memory of things precious keepeth warm
The heart that once did hold them. —*J. Ingelow.*

What need to look behind thee and to sigh?
When God left speaking, He went on before
To draw men after, following up and on;
And thy heart fails because thy feet are slow!
Thou think'st of Him as one that will not wait.
A Father and not wait! He waited long
For us, and yet, perchance, He thinks not long
And will not count the time. There are no dates
In His fine leisure! —*J. Ingelow.*

O Lord, my God, do Thou Thy holy will!
I will be still;
I will not stir lest I forsake Thine arm,
And break the charm
Which lulls me, clinging to my Father's breast,
In perfect rest! —*Keble.*

**Reading.**

When thou passest through the waters I will be with thee; and through the rivers, they shall not overflow thee; when thou walkest through the fire thou shalt not be burned; neither shall the flame kindle upon thee.—*Isaiah xliii.* 2.

They may rise very high, these waters of affliction, these rivers of trouble, but this promise assures us that they shall not overwhelm me. God assures me of this; let that be enough. In very great pain, in sore discomfort, in deep depression of spirit, I do sometimes feel almost overwhelmed, and as if I could not bear any more. Then more will not come, for all is in the hand of God. And He Himself tells me that the rivers shall *not* overflow me; that my sorrows and sufferings shall not become too great. O Lord my God, thou knowest all my ways, all that happens to me; thou knowest what particular time I am passing through the waters; thou knowest how deep they are; thine eye is upon me always. Thou seest me. Thou knowest what I suffer, what I think, what I fear. I do venture, humbly, oh, my Father, to draw near to Thee as my refuge in this time of trouble. "I will be with thee." I hear Thy gracious words, and I come to Thee my Saviour, Jesus Christ, in whom is all my trust, and in Whom "Thou art always well pleased." Cause me to feel that Thou *art* near, my Father, then nothing shall terrify me. Nothing shall cast me down. "Yea, though I walk through the valley of the shadow of death I will fear no evil, for Thou art with me. Thy rod and Thy staff they comfort me."—*Francis Bourdillon.*

### Notes and Queries.

*Allowances Under Poor Law Authorities.*

(172) I have been nursing for nearly 20 years, and am now 40 years old, and my health is not very good. I have been working under the Poor Law Authorities for eleven years, and have a two years' certificate. Will you kindly tell me if I am entitled to any allowance, and the name of the proper person to whom to apply?—*Anxious.*

If you are now in the service of the guardians, and can obtain medical proof of incapacity for work, you are certainly entitled to a pension. You must apply to the guardians under whom you are working.

*A District Nurse's Duty.*

(173) Is a district nurse exceeding her duty by going in to see a patient when asked to by the people when they saw her passing? She sent for the doctor, who said she ought not to go to any patient unless they first sent for him, and he would send for her. The question is of moment to district nurses in general, as often in country districts the doctor is not easily got at short notice, and the nurse may alleviate much suffering in the meantime, and even save life, as in case of hæmorrhage.—*A. P.*

The only right that a nurse has to enter any house is that she is asked to do so by the occupier of that house. So long as a nurse acts honourably and does not attempt to undertake any medical treatment, or to give any advice beyond what is part of mere nursing, we cannot see that a medical man has any cause of complaint. It certainly is not true as a general thing that the nurse is sent by the medical man.

*Weak Intellect.*

(174) Could you let me know the name of any school or home for the reception of a little boy of weak intellect?—*W. H. Knighton.*

Children of weak intellect should be placed under special instructions at as early an age as possible, as very little can be done for them in later years. The two national asylums are the Royal Albert Asylum for Idiots and Imbeciles, Lancaster (free and paying patients admitted), and the Earlswood Asylum for Idiots and Imbeciles, Redhill (office, 36, King William Street, E.C.)

*Private Home.*

(175) Will you very kindly notice in THE HOSPITAL that I have taken a private nursing home for paying patients?—*E. P. H.*

Our rules forbid us to notice any home unless it be managed by a committee, or we have been able to inspect it personally.

*A Young Probationer.*

(176) I have a sister living in Wales, age 20, who wishes to become a nurse. Is she old enough, and what steps must she take to obtain an appointment?—*J. W.*

She is old enough to enter a children's hospital, where she gains experience, though the time spent in such hospitals does not count for a certificate. See Honnor Morten's "How to Become a Nurse," price 2s. 6d., of the Scientific Press, 28 & 29, Southampton Street, Strand.

*L.O.S. Examination Questions.*

(177) Will you kindly inform me where I can obtain the last examination questions of the Obstetric Society?—*Meta.*

At the Midwives' Institute, 12, Buckingham Street, Strand.

*Training as Cottage Nurse.*

(178) Could you give me any information as to where I could have a young woman trained for six months as cottage nurse for a country district?—*T. Symes.*

The Maternity Charity, Plaistow, E.

*Spelling.*

(179) A friend of mine who is well educated, but spells badly, wants to know if this defect would be a drawback in a nurse's career.—*Bet.*

A slight one only. It would tell a little against her in examinations; that is all. The art of nursing is quite independent of the art of spelling.

*Training for L.O.S.*

(180) Which is the best hospital in London where full training in midwifery for the L.O.S. certificate, with or without fee, can be obtained?—*A Nurse.*

No hospital trains without a fee. Queen Charlotte's Hospital, Marylebone Road; the General Hospital, York Road; the East London Mothers' Home; the Clapham Maternity Hospital, &c., all give excellent training at various fees, the East London Mothers' Home being the cheapest.

*Nursing in Africa.*

(181) If it is not too late, to whom must I apply in order to accompany Major Lugard's expedition to Africa as nursing sister?—*Nurse Lena.*

The nurses who accompany Major Lugard are from the London Hospital. As the expedition starts at the end of this week it is certainly too late to apply.

*Notification.*

(182) Will you tell me if public health authorities consider it necessary to fumigate poor people's houses after typhoid fever? And if so, whose duty is it to report the convalescence of the patient?—*Rachel.*

In most districts the case will have been notified to the medical officer of health by the doctor in attendance, but the convalescence should be reported by the head of the family, or by the nearest relatives, or by the nurse. The sanitary authority will take measures to ensure what is necessary being done.

*Home for Incurable Consumptive Patients.*

(183) *Nurse H.* should write to the Assistant Superior, St. Peter's Home, Mortimer Road, Kilburn, N.W. Incurable consumptive cases (women) are received there.

